# Self-Supply Oxygen ROS Reactor via Fenton-like Reaction and Modulating Glutathione for Amplified Cancer Therapy Effect

**DOI:** 10.3390/nano12142509

**Published:** 2022-07-21

**Authors:** Huanli Zhang, Wei Ma, Zhiqiang Wang, Xiaodan Wu, Hui Zhang, Wen Fang, Rui Yan, Yingxue Jin

**Affiliations:** Key Laboratory for Photonic and Electronic Bandgap Materials, Ministry of Education, College of Chemistry & Chemical Engineering, Harbin Normal University, Harbin 150025, China; hxzhl0923@163.com (H.Z.); maweimcz@163.com (W.M.); wzq70402@163.com (Z.W.); smile_200325@163.com (X.W.); huihui279727690@163.com (H.Z.)

**Keywords:** reactive oxygen species (ROS), Fenton-like reaction, self-supply oxygen, tumor microenvironment (TME), synergetic effect

## Abstract

Reactive oxygen species (ROS) are highly reactive oxidant molecules that can kill cancer cells through irreversible damage to biomacromolecules. ROS-mediated cancer therapies, such as chemodynamic (CDT) and photodynamic therapy (PDT), are often limited by the hypoxia tumor microenvironment (TME) with high glutathione (GSH) level. This paper reported the preparation, characterization, in vitro and in vivo antitumor bioactivity of a meso-tetra(4-carboxyphenyl)porphine (TCPP)-based therapeutic nanoplatform (CMMFTP) to overcome the limitations of TME. Using Cu^2+^ as the central ion and TCPP as the ligand, the 2D metal-organic framework Cu-TCPP was synthesized by the solvothermal method, then CMMFTP was prepared by modifying MnO_2_, folic acid (FA), triphenylphosphine (TPP), and poly (allylamine hydrochloride) (PAH) on the surface of Cu-TCPP MOFs. CMMFTP was designed as a self-oxygenating ROS nanoreactor based on the PDT process of TCPP MOFs and the CDT process by Cu(II) and MnO_2_ components (mainly through Fenton-like reaction). The in vitro assay suggested CMMFTP caused a 96% lethality rate against Hela cells (MTT analysis) in specific response to TME stimulation. Moreover, the Cu(II) and MnO_2_ in CMMFTP efficiently depleted the glutathione (80%) in tumor cells and consequently amplified ROS levels to improve CDT/PDT effects. The FA-induced tumor targeting and TPP-induced mitochondria targeting further enhanced the antitumor activity. Therefore, the nanoreactor based on dual targeting and self-oxygenation-enhanced ROS mechanism provided a new strategy for cancer therapy.

## 1. Introduction

Malignant neoplasms are one of the most dreaded diseases due to their high mortality and difficulty in curing [1]. The tumor microenvironment (TME), characterized by hypoxia, high expression of glutathione (GSH), weak acidity, and high concentration of H_2_O_2_, is closely related to the immunosuppression, multidrug resistance and aggressive metastasis of cancer cells, and has become a new target for cancer therapy [2,3]. Reactive oxygen species (ROS) are highly oxidizing species such as hydroxyl radicals, singlet oxygen, superoxide radicals, and hydrogen peroxide. ROS can kill cancer cells by irreversibly damaging DNA, RNA, proteins, and other biological macromolecules through oxidation reactions [4,5]. ROS-mediated therapies such as chemodynamic (CDT) and photodynamic therapy (PDT) have become a new promising cancer treatment method [6,7,8,9]. However, PDT/CDT are often limited by the insufficient concentration of O_2_ and H_2_O_2_ in TME [10,11,12], and also by the disruption of the redox homeostasis system in tumor cells [13]. Many ROS-mediated chemotherapeutic agents such as paclitaxel and DOX are not selective and have greater damage to normal tissue [14]. Therefore, overcoming the limitations of TME and improving the specific selectivity are urgent issues for ROS-mediated therapy.

CDT is a highly selective ROS-mediated cancer therapy developed in recent years [15,16,17,18]. CDT can convert endogenous H_2_O_2_ in TME into ^•^OH and O_2_ to alleviate TME hypoxia [19,20,21]. Because O_2_ is produced endogenously, there is no need to concern about the additional risk in externally O_2_-delivery process. Therefore, CDT has offered hope for selective treatment and alleviation of TME hypoxia. However, the insufficient concentration of H_2_O_2_ in TME limits the practical application [22,23]. PDT is also a ROS-mediated therapy with spatio-temporal selectivity in response to localized specific laser stimulation [24], which generates highly cytotoxic ROS (^1^O_2_) by transferring light energy to surrounding O_2_ through localized light radiation. However, PDT is heavily dependent on tissue oxygen concentration. TME hypoxia severely limits the clinical application of PDT. Fortunately, in situ oxygen production by CDT can supplement the TME oxygen concentration. Therefore, synergistic CDT and PDT treatment is a very promising strategy to overcome the limitations of TME. Another important barrier to ROS-mediated therapy is the high expression of GSH in TME. GSH is extremely sensitive to exogenous ROS and can rapidly consume ROS through redox reactions to maintain intracellular redox homeostasis. Therefore, the GSH is regarded as a debilitating factor for CDT and PDT, and overcoming ROS depletion by GSH is a serious challenge for CDT and PDT [25,26,27,28].

Meso-tetra(4-carboxyphenyl)porphine (TCPP) is a commonly used PDT photosensitizer with strong absorption and fluorescence emission in the near infrared. The four carboxyl groups in TCPP can easily form metal organic frameworks (MOFs) with transition metal ions. MOFs are crystalline porous materials with periodic network structure formed by self-assembly of transition metal ions and organic ligands, which have the advantages of high porosity, large specific surface area, regular pore channel and adjustable pore size. MOFs have been widely used in electronics, environmental, and energy fields. In recent years, MOFs have also shown their advantages in the biomedical field and become a new promising drug carrier [29,30,31,32].

**Scheme 1:**
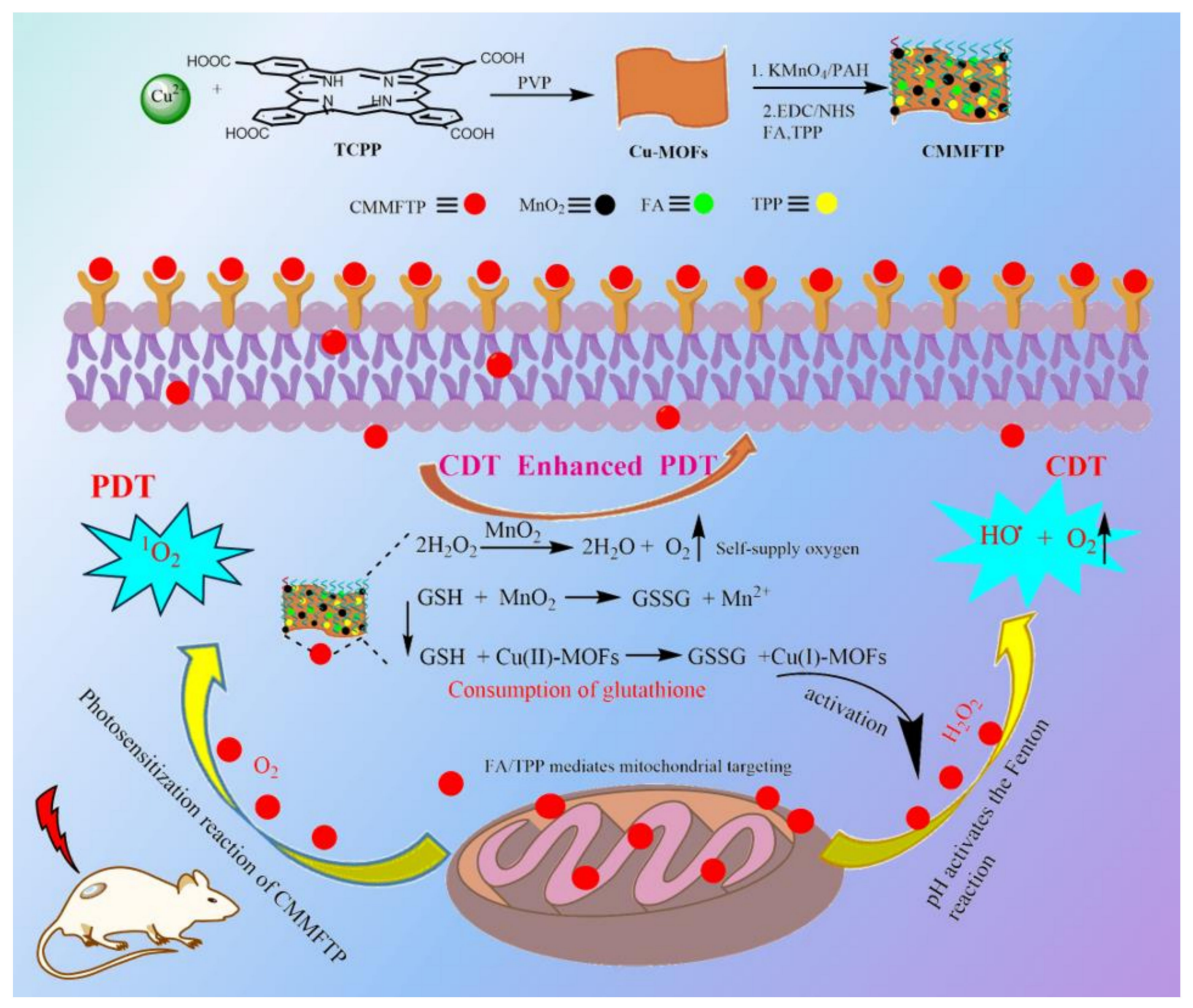
The synthetic route and anti-tumor mechanism of a TME-responsive self-oxygenation-enhanced nanoreactor (CMMFTP) based on CDT/PDT synergy and GSH depletion strategy.

In this study, a TME-responsive self-oxygenation-enhanced nanoreactor, Cu-TCPP MOFs@MnO_2_/FA/TPP@PAH (abbreviated as CMMFTP), was prepared based on CDT/PDT synergy and GSH depletion strategy. The CDT of Cu-MOF and MnO_2_ synergize with the PDT of TCPP to enhance the anti-tumor effects, and the tumor targeting of FA and the mitochondrial targeting of TPP result in precise anchoring of CMMFTP to mitochondria [33,34,35,36]. Copper and manganese ions in CMMFTP can inhibit ROS consumption by GSH and amplify oxidative stress [21,37,38,39,40,41,42]. The synthesis and therapy mechanism of CMMFTP are shown in Scheme 1.

## 2. Results and Discussion

### 2.1. Preparation and Characterization

Cu-TCPP MOF (Cu-MOFs) were prepared by hydrothermal synthesis, with Cu^2+^ as the central atom, TCPP as the ligand, and polyvinyl pyrrolidone (PVP) as the crystal anisotropic growth inducer to induce the lateral growth of MOF crystals. Poly(allylamine hydrochloride) (PAH) was used as a reducing agent [43] to react in situ with KMnO_4_ on the surface of Cu-MOFs to generate MnO_2_, and then MnO_2_ was electrostatically loaded on Cu-MOFs to obtain Cu-MOFs@MnO_2_, and the unreacted PAH on the surface was removed by repeated washing with ethanol and water. The surface of Cu-MOFs is rich in carboxyl groups, which were activated by 1-ethyl-3-(3-dimethylaminopropyl) carbodiimide hydrochloride (EDC) and N-hydroxysuccinimide (NHS), and then modified with FA and TPP to obtain dual-targeting nanoparticles Cu-MOFs@MnO_2_@FA/TPP. Then PAH was added to form biocompatible Cu-MOFs@MnO_2_@FA/TPP@PAH (CMMFTP) by electrostatic adsorption on the surface.

**Figure 1:**
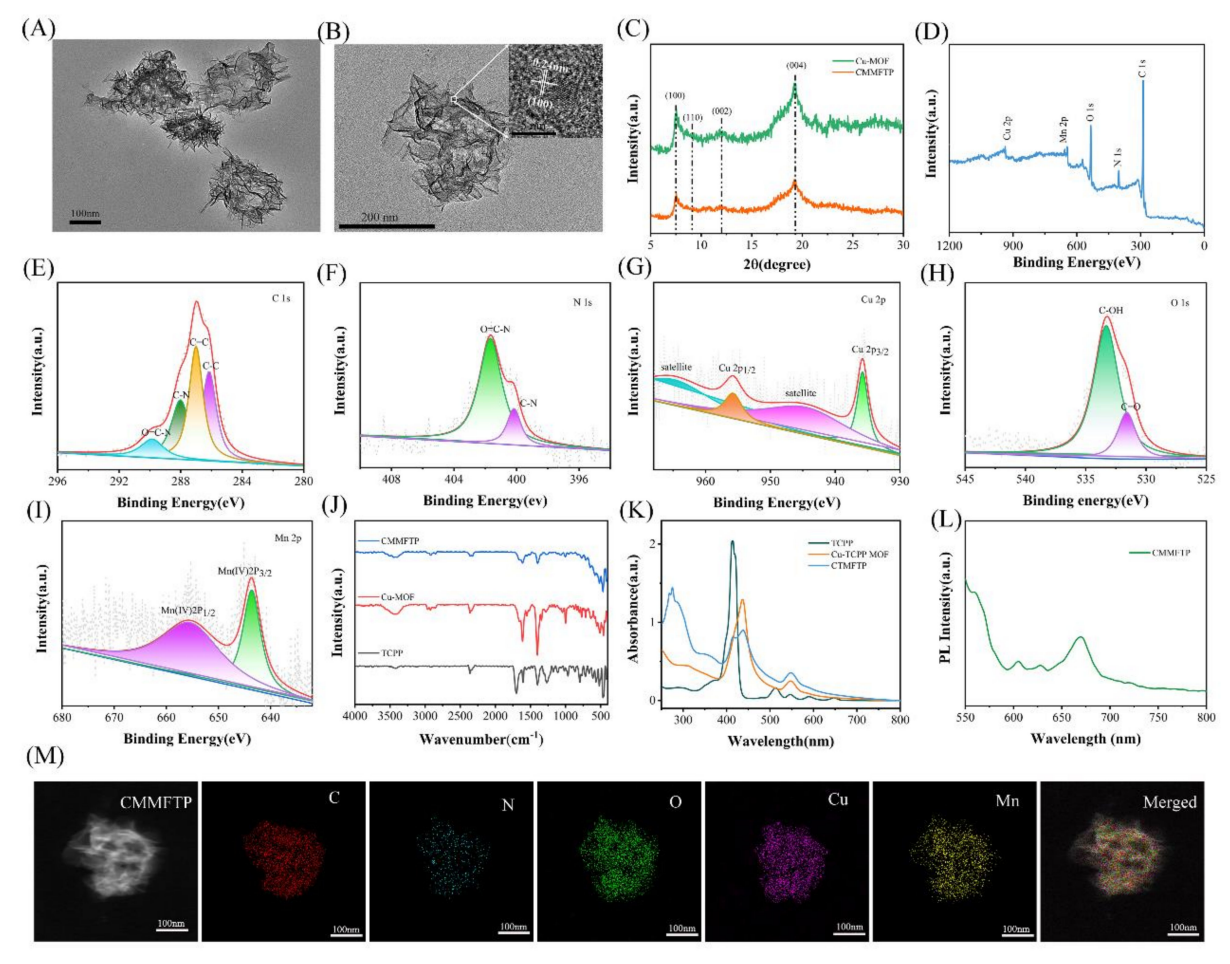
(**A**) Transmission electron microscopy (TEM) image of CMMFTP; (**B**) high-resolution transmission electron microscope (HR-TEM) image of CMMFTP (Inset: HR-TEM image of MnO_2_ nanocrystals); (**C**) X-ray diffraction (XRD) patterns of CMMFTP; (**D**) X-ray photoelectron spectroscopy (XPS) spectra of CMMFTP; (**E**) high-resolution X-ray photoelectron spectroscopy (HR-XPS) of C 1s; (**F**) HR-XPS of N 1s; (**G**) HR-XPS of Cu 2p; (**H**) HR-XPS of O 1s; (**I**) HR-XPS of Mn 2p; (**J**) Fourier transform infrared (FT-IR) spectra of TCPP, Cu-MOFs and CMMFTP; (**K**) UV-visible absorption spectra of TCPP, Cu-MOFs and CMMFTP; (**L**) Fluorescence emission spectrum of CMMFTP; (**M**) dark-field TEM image and corresponding elemental mappings images of CMMFTP.

Figure 1A shows the transmission electron microscope image of CMMFTP, which clearly shows its morphology as a rectangular sheet with a long side size of approximately 210 nm. And as seen in Figure S1, the hydrodynamic diameter of CMMFTP measured by dynamic light scattering (DLS) was 230 ± 30 nm (in Supplementary Material). The high-resolution transmission electron microscope image (Figure 1B) showed that the CMMFTP was an ultra-thin sheet, and the embedded image in the upper right corner clearly shows the lattice stripe spacing d = 0.24 nm of MnO_2_ nanocrystals, indicating that MnO_2_ was successfully loaded on Cu-MOFs. Figure 1C shows the XRD patterns of CMMFTP. The surface modification of Cu-MOFs with MnO_2_, FA, TPP, PAH did not change the crystalline shape, indicating that the crystals were not destroyed during the surface carboxyl activation and condensation reaction of Cu-MOFs@MnO_2_, and the structure of Cu-MOFs was also stable after long time reaction in polar reaction solvent. Figure 1D showed the X-ray photoelectron spectroscopy (XPS) of CMMFTP, which suggested that CMMFTP consisted of five elements: C, N, O, Mn, and Cu. Figure 1E–I shows the high-resolution XPS (HR-XPS) of C, N, Cu, O, and Mn, respectively. Figure 1G shows that Cu was divalent, and 935.2 eV and 955.2 eV were attributed to Cu(II)2p_3/2_ electron diffraction peak and Cu(II)2p_1/2_ electron diffraction peak, respectively, and 942.9 eV and 962.7 eV were Cu(II) oxygen peaks, indicating that Cu-MOFs were successfully constructed and the central node of Cu(II) ions was stable.

Figure 1I shows the HR-XPS of Mn with two electron diffraction peaks spaced by 11.7 eV, which is consistent with the typical Mn^4+^ electron diffraction peak characteristics [33], indicating the successful loading of MnO_2_ on Cu-MOFs. Figure 1J was the Fourier transform infrared (FT-IR) spectra, and there were Cu-O vibrational peaks at 600 cm^−1^ and Mn-O vibrational peaks at 520 cm^−1^, which again demonstrated the loading of MnO_2_ on Cu-MOFs. The O-H vibrational peaks at 3250–3550 cm^−1^ indicated the presence of carboxyl groups on the surface of Cu-TCPP MOFs. Figure 1K showed the UV−visible absorption spectra of TCPP, Cu-MOFs, and CMMFTP. The peak shape of Cu-MOFs was consistent with that of TCPP, but the characteristic absorption peak was slightly red-shifted, which was obviously due to the complexation of TCPP with Cu(II). Fluorescence emission spectrum of CMMFTP is shown in Figure 1L, with a strong emission peak at 680 nm (λex 410 nm). The high-angle annular dark-field scanning TEM image and corresponding elemental mappings of CMMFTP are shown in Figure 1M, showing the presence of C, N, O, Cu and Mn elements, which is consistent with the XPS results.

The above analysis indicated the successful preparation of CMMFTP. Figure S2 (in Supplementary Material) showed the zeta potential potentials of Cu-MOFs, Cu-MOF@MnO_2_, Cu-MOF@MnO_2_@FA/TPP, CMMFTP. The potential of Cu-MOFs was −18.55 mV, indicating a large number of negatively charged carboxyl groups on the surface, and increased to −5.82 mV after modification of TPP. The surface completely converted to positive charge after PAH-ization, reaching 8.63 mV. To examine whether CMMFTP is stable in complex physiological environments, the particle size changes in saline, PBS (pH 7.4), and DMEM medium were examined. As shown in Figure S3 (in Supplementary Material), there was no turbidity of the solution and no precipitate generation was observed during the 15 days, and the particle size of CMMFTP remained essentially unchanged, indicating stability in complex environments.

### 2.2. Performance Analysis

#### 2.2.1. Regulation of the Production ^•^OH by pH and GSH

**Figure 2:**
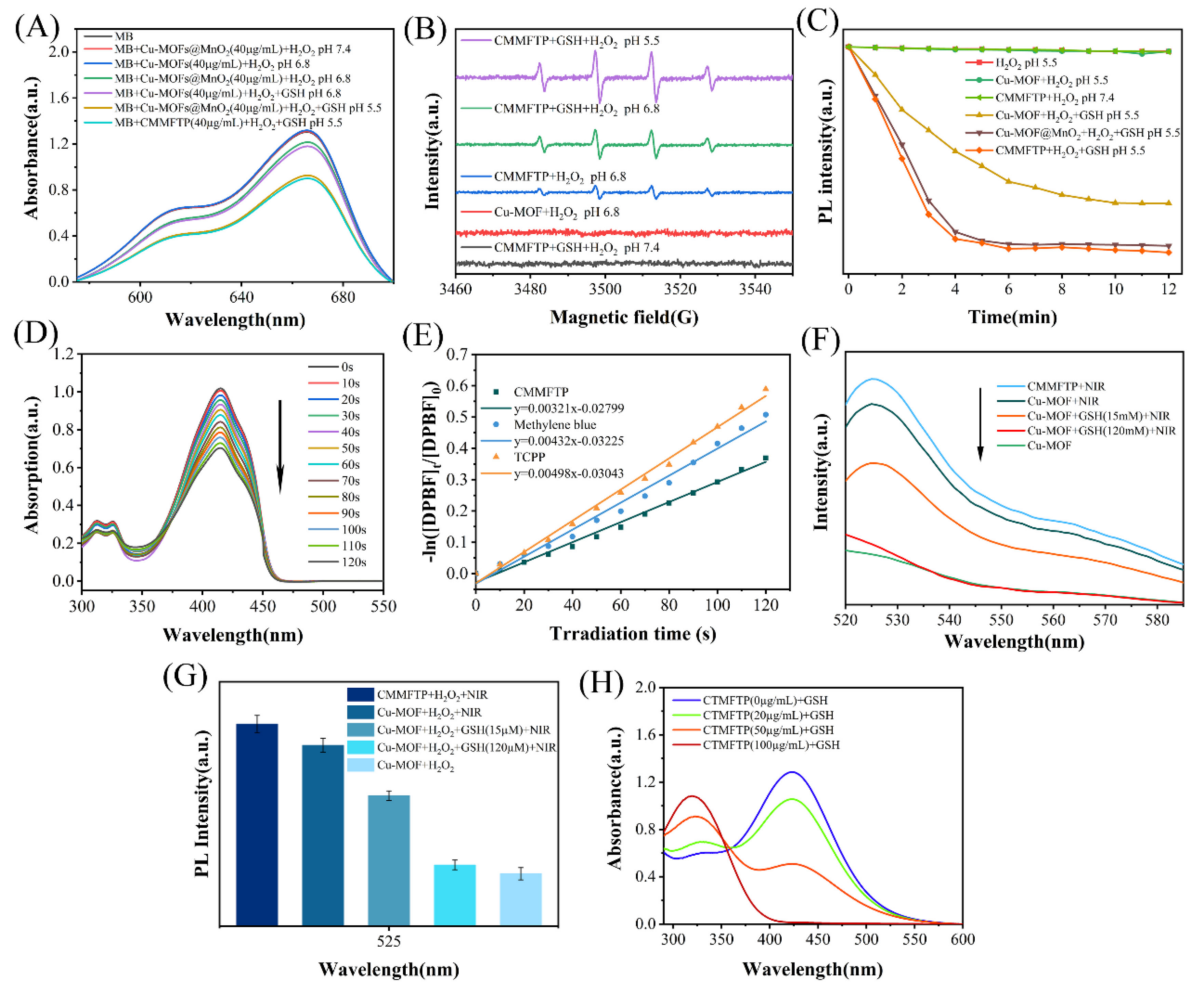
(**A**) Detection of ^•^OH generation capacity of various samples using MB; (**B**) ^•^OH detection by ESR signals under different conditions; (**C**) detection of O_2_ production capacity of various samples using RDPP as a dissolved oxygen probe; (**D**) examine the ability of CMMFTP photosensitization to produce ^1^O_2_ using DPBF as singlet oxygen indicator probe; (**E**) standard curves of degradation rate and light time of DPBF; (**F**) fluorescence spectra and (**G**) fluorescence intensity histograms of ^1^O_2_ detected with the SOSG probe; (**H**) detection of GSH consumption by CMMFTP with DTNB probe.

Cu^2+^ and MnO_2_ are excellent Fenton-like reaction reagents, reacting with hydrogen peroxide to form ^•^OH. In this study, we examined the ability of the Fenton-like reaction of CMMFTP to generate ^•^OH using methylene blue (MB) as a ^•^OH trapping agent and investigated the influence of pH value and GSH on the Fenton-like reaction of CMMFTP [44]. The absorbance of MB at 650 nm was negatively correlated with the concentration of ^•^OH. In the experiments, the concentration of H_2_O_2_ was 30 µg/mL and the concentration of Fenton-like reagent was 40 µg/mL. Figure 2A showed that there was no ^•^OH production in the Cu-MOFs@MnO_2_ + H_2_O_2_ pH 7.4 sample, indicating that neither Cu-MOFs nor MnO_2_ underwent Fenton-like reaction in weak alkalinity. No ^•^OH was produced in the Cu-MOFs + H_2_O_2_ pH 6.8 sample, indicating that the Cu(II)-MOFs constructed with divalent copper ions did not undergo Fenton-like reaction even in weak acidity. While Cu-MOFs + H_2_O_2_ + GSH pH 6.8 sample showed ^•^OH production, indicating that GSH initiated the Fenton-like reaction of Cu(II)-MOFs: GSH reduced Cu(II)-MOFs into Cu(I)-MOFs, and the Cu(I) paddlewheel complexes start the Fenton-like reaction, during which hydrogen peroxide was decomposed into ^•^OH.

Comparing Cu-MOFs + H_2_O_2_ + GSH pH 6.8 samples with Cu-MOFs@MnO_2_ + H_2_O_2_ pH 6.8 samples, the latter produced more ^•^OH, indicating that MnO_2_ also underwent Fenton-like reaction. The dual Fenton-like reaction of Cu-MOFs and MnO_2_ synergistically enhanced the ^•^OH level of CMMFTP. Moreover, the ^•^OH level of Cu-MOFs@MnO_2_ + H_2_O_2_ pH 5.5 samples further increased, indicating that the Fenton-like reaction of Cu-MOFs@MnO_2_ was enhanced with increasing acidity. The CMMFTP + H_2_O_2_ + GSH pH 5.5 samples were comparable to the Cu-MOFs@MnO_2_ + H_2_O_2_ + GSH pH 5.5 samples in terms of ^•^OH production efficiency, indicating that the loaded small molecules (FA, TPP) and macromolecules (PAH) did not decrease the Fenton-like reaction capacity of CMMFTP. The amount of ^•^OH was proportional to the concentration of CMMFTP (0–80 µg/mL) (Figure S4).

The influence of pH and GSH on Fenton-like reaction of CMMFTP to produce ^•^OH was further verified by electron spin resonance (ESR) experiments (Figure 2B). 5,5-Dimethyl-1-pyrroline N-oxide (DMPO) was used as a ^•^OH trapping agent, and four signal peaks with an intensity of 1:2:2:1 appeared in the ESR spectrum after trapping by DMPO. It was found that CMMFTP did not undergo Fenton-like reaction under alkaline conditions, even in the presence of GSH. The absence of ^•^OH signal in the Cu-MOFs + H_2_O_2_ pH 6.8 sample indicated that the Fenton-like reaction did not occur in divalent copper at this time. On the contrary, there was a clear ^•^OH signal peak in the CMMFTP + H_2_O_2_ pH 6.8 sample, indicating that the ^•^OH signal peak was generated by the Fenton-like reaction of MnO_2_. The signal of CMMFTP + GSH + H_2_O_2_ sample was further enhanced, indicating that GSH initiated the Fenton-like reaction of Cu-MOF. The strongest signal peak was observed for the CMMFTP + GSH + H_2_O_2_ pH 5.5 sample, indicating that the Fenton-like reaction of CMMFTP was enhanced with increasing acidity. The MB capture experiments were consistent with the results of ESR experiments, and both demonstrated the ability of pH and GSH to modulate the production of ^•^OH by CMMFTP.

#### 2.2.2. Regulation of the Production O_2_ by pH and GSH

The Fenton-like reaction can generate oxygen to relieve TME hypoxia and replenish oxygen for PDT [45]. The effect of CMMFTP-responsive O_2_ generation from H_2_O_2_ was measured using the commercial chemical probe [Ru(dpp)_3_]Cl_2_ (RDPP). Molecular oxygen causes a significant reduction in the fluorescence of RDPP due to the dynamic quenching, and the fluorescence intensity (λ_ex_ 455 nm/λ_em_ 613) is negatively correlated with O_2_ concentration. Thus, O_2_ was detected by measuring the fluorescence intensity. The samples to be tested were preemptively evacuated of dissolved oxygen with a nitrogen stream and followed by the addition of RDPP. Subsequently, H_2_O_2_ was added and the fluorescence intensity was recorded. As shown in Figure 2C, the fluorescence intensity of RDPP did not change in pure H_2_O_2_ solution, indicating that the fluorescence of RDPP was not altered by H_2_O_2_. The Cu-MOFs pH 5.5 samples did not produce oxygen, while Cu-MOFs + GSH pH 5.5 samples produced oxygen obviously, indicating that Cu(II)-MOF does not undergo Fenton-like reaction in GSH-free samples, and can produce oxygen only after being activated by GSH. This result was consistent with the results of MB capture ^•^OH and ESR experiments (Figure 2A,B), which again demonstrated the ability of GSH to regulate the Fenton-like reactions of Cu-MOFs to produce O_2_ and ^•^OH. Comparing the oxygen yields of Cu-MOFs + GSH pH 5.5 samples and Cu-MOF@MnO_2_ + GSH pH 5.5 samples, the latter was significantly higher than the former, which was obviously the result of the catalytic decomposition of H_2_O_2_ based on the synergistic CDT process of MnO_2_ and Cu-MOFs. The oxygen production capacity of CMMFTP was comparable to that of Cu-MOFs@MnO_2_, indicating that modification of functional small molecules (FA, TPP) and macromolecules (PAH) on the surface did not affect the oxygen production capacity. Therefore, O_2_-capture experiments by RDPP demonstrated that the Fenton-like reactions of CMMFTP could be regulated by pH and GSH (weak acidity and GSH stimulation), indicating that the Fenton-like reactions of CMMFTP are specifically selective and no Fenton-like reactions occur in weakly alkaline (pH 7.4) solutions, also making tumor-specific selective treatment possible.

#### 2.2.3. Production of Singlet Oxygen(^1^O_2_)

TCPP absorbs in the near-red light region and is a commonly used PDT photosensitizer. The absorption wavelength was slightly red-shifted after the complexation with Cu(II), and the spectral properties did not change after the loading of MnO_2_, FA/TPP and surface modification of PAH (Figure 1K). The quantum yield of ^1^O_2_ is an important indicator for PDT photosensitizers. In this study, 1,3-diphenylisobenzofuran (DPBF) was used as a singlet oxygen trapping agent to examine the ability of CMMFTP photosensitization to produce ^1^O_2_, and the quantum yield of ^1^O_2_ was calculated with MB as a reference. After adding DPBF, the CMMFTP and MB samples were irradiated with laser (660 nm, 50 mW/cm^2^) to generate ^1^O_2_, the absorption peak at 412 nm was monitored (Figure 2D). The degradation rate of DPBF was linearly and positively correlated with the light exposure time (Figure 2E). The ^1^O_2_ quantum yield of CMMFTP was 36.92% according to Equation (1) (the specific experimental data were listed in Table S1) (Φ was the quantum yield of singlet oxygen, T was the illumination time), which was smaller than that of TCPP, presumably due to the partial energy transfer from the excited state of Cu-TCPP MOFs to Cu(II) and MnO_2_.

(1) $$\Phi_{s}=\Phi_{\mathrm{MB}}\;\frac{Ts}{T_{\mathrm{MB}}}$$

Singlet Oxygen Sensor Green (SOSG) is also a widely used singlet oxygen fluorescent probe [35], which is non-fluorescent itself and oxidized by ^1^O_2_ to produce SOSG-O, and can emit green fluorescence (Ex/Em:504/525 nm) that is positively correlated with the amount of ^1^O_2_. Therefore, the amount of ^1^O_2_ can be indirectly detected by monitoring the fluorescence intensity. Figure 2F showed the normalized fluorescence intensity of SOSG-O under different conditions, and Figure 2G showed the histogram of normalized fluorescence intensity, which indicated that Cu-MOFs did not generate ^1^O_2_ in the dark, yet could produce singlet oxygen after illumination. The CMMFTP was more capable of producing ^1^O_2_ than Cu-MOFs, which indicated that the surface modifications (FA, TPP, PAH) increased the dispersion of Cu-MOFs, reduced the fluorescence burst caused by aggregation, and thus increased the quantum yield of ^1^O_2_. The research also demonstrated that PAH is beneficial not only for increasing the biocompatibility of the material, but also for increasing the yield of ^1^O_2_ by reducing aggregation. According to the above discussion, CMMFTP is an intelligent and specific response of ROS generator for cancer treatment.

#### 2.2.4. Depletion GSH

GSH is a serious barrier to ROS-mediated treatment of cancer, and Cu(II) and MnO_2_ are known as excellent inhibitors of GSH, so the central Cu(II) in CMMFTP and the loaded MnO_2_ will act synergistically to inhibit GSH. In this paper, the ability of CMMFTP to deplete GSH was examined using 5,5-dithiobis(2-nitrobenzoic acid) (DTNB) (Figure 2H). DTNB absorbs at 325 nm and can be reduced by GSH to form 2-nitro-5-thiobenzoic acid (NTB), and the absorbance of NTB at 412 nm is proportional to GSH consumption. As shown in Figure 2H, the absorption peak at 412 nm gradually decreased as the concentration of CMMFTP increased, while the absorption peak at 325 nm gradually increased. Moreover, the intensity of the peak at 412 decreased obviously at a CMMFTP concentration of 100 µg/mL (the peak at 325 nm was the largest at this time), indicating that a large amount of GSH was consumed, and the consumption of GSH was positively correlated with the CMMFTP concentration. Therefore, CMMFTP had a strong ability to consume GSH, consequently could amplify the ROS level.

### 2.3. In Vitro Studies

#### 2.3.1. Evaluation of Cellular Uptake and Targeting of CMMFTP

The lifespan of ROS is extremely short, so the ROS-mediated therapeutic drugs must enter the cell effectively. The specific targeting of drugs can facilitate drug entry into cancer cells, reduce damage to normal tissues, reduce the drug dose and decrease side effects. The tumor cell targeting molecule FA and mitochondrial targeting molecule TPP on CMMFTP confer dual targeting functions. Therefore, CMMFTP is expected to have excellent target aggregation function and precise mitochondrial anchoring ability. In this study, the targeting function and cellular uptake ability of CMMFTP were examined using folic acid receptor overexpressing Hela and 4T1 cells as model cells. CMMFTP itself emits red fluorescent properties after irradiation and can be directly observed under fluorescence microscope without fluorescent markers.

**Figure 3:**
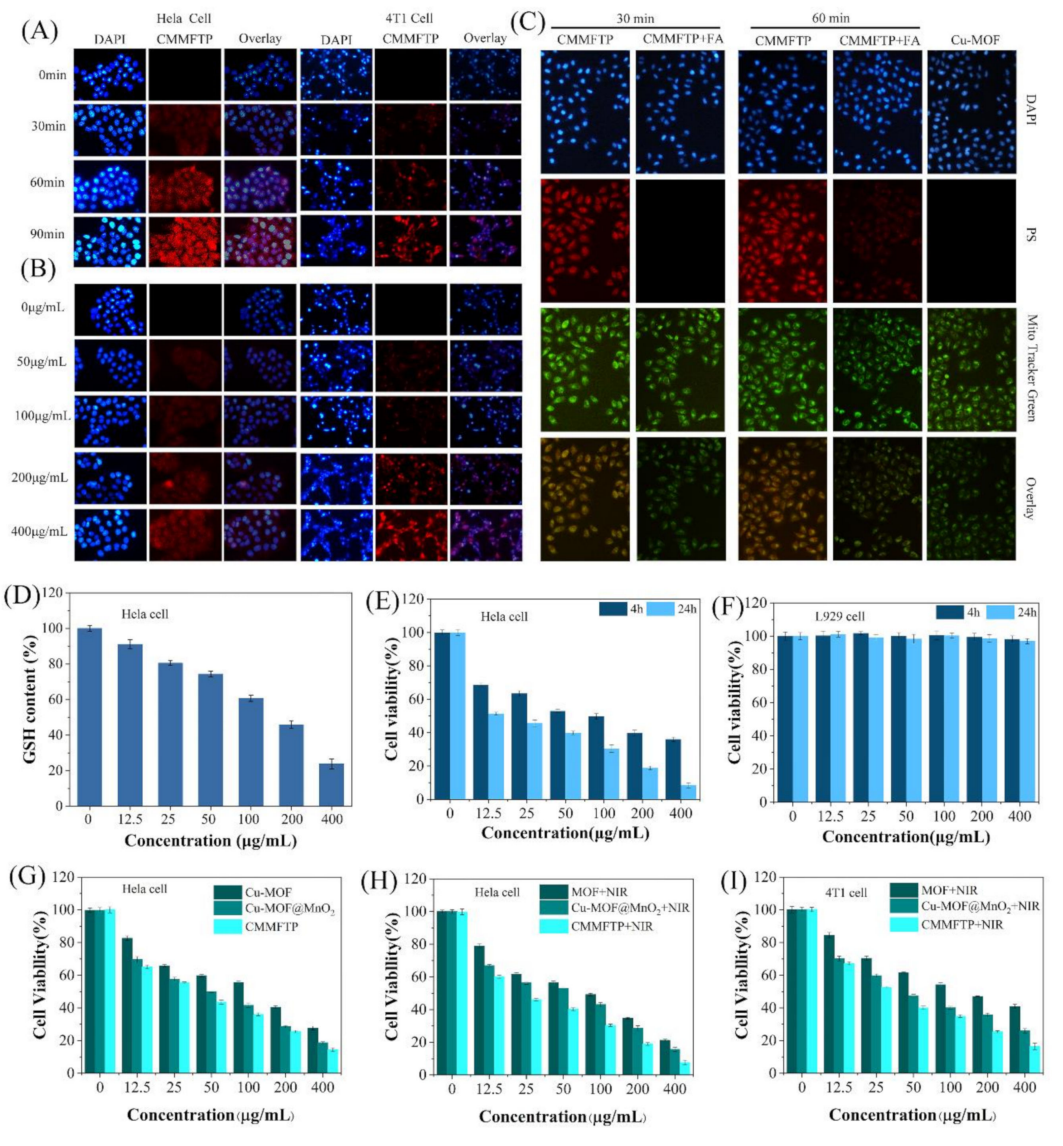
(**A**) Time (100 µg/mL, 0–90 min) dependence and (**B**) concentration (30 min, 0–400 µg/mL) dependence images of cell uptake; (**C**) detection of mitochondrial targeting of CMMFTP with Mito-Tracker Green; (**D**) detection of the intracellular GSH depletion capacity of CMMFTP; (**E**) dark toxicity of CMMFTP in Hela cells; (**F**) dark toxicity of CMMFTP in normal cells; (**G**–**I**) comparison of the cytotoxicity of Cu-MOFs, Cu-MOF@MnO_2_, CMMFTP under different conditions. *p* < 0.05.

Hela cells and 4T1 cells were treated with CMMFTP (100 µg/mL) and incubated in the incubator for 0–90 min, and the nuclei were stained with DAPI and observed under a fluorescent inverted microscope. The results in Figure 3A showed that there was stronger red fluorescence in Hela cells and 4T1 cells at 60 min, indicating that a large amount of CMMFTP entered the cells, and the red fluorescence in Hela cells was stronger, indicating that CMMFTP was more easily taken up by Hela cells. The red fluorescence was stronger after 90 min incubation, indicating that CMMFTP was uptaken by cells in a time-dependent manner. Then, the cells treated with different concentrations of CMMFTP (0–400 µg/mL) were observed at 30 min (Figure 3B). As the drug concentration increased, intracellular red fluorescence increased, and the cell uptaking was also concentration-dependent.

To examine tumor-specific targeting, cells were first treated with CMMFTP (200 µg/mL) and with or without folic acid (20 μg/mL) and incubated in the incubator, then treated with mitochondrial probe Mito-Tracker Green (which could be used for mitochondria-specific fluorescent staining of living cells). Figure 3C showed the results of targeting experiments. In the folic acid-treated cell groups, there was no red fluorescence of CMMFTP at 30 min, indicating that almost no CMMFTP entered the cells, which suggested the pre-added FA first occupied the FA receptor on the cell membrane and blocked the binding of nano-drug to the receptor. The weak red fluorescence of mitochondrial probe at 60 min indicated that only a small amount of nanodrug entered the cells, indicating that the cell membrane surface screened off CMMFTP after binding to folic acid, which prevented the drug from binding to the membrane and entering the cells. In contrast, in the non-folic acid-treated cell groups, strong red fluorescence of CMMFTP overlapped with the green fluorescence in cells after 30 min, indicating that a large amount of CMMFTP entered the cells. The ratio of overlap increased with prolonged time, indicating that the folic acid of CMMFTP binds to the folic acid receptors on the cell membrane surface, which facilitated the internalization of CMMFTP, also verified the specific targeting of CMMFTP. Meanwhile, the increased overlapping ratio of red and green fluorescence suggested that nano-drug has gradually anchored to the mitochondria by electrostatic induction of the negative charge on the mitochondrial membrane, suggesting the specific targeting to mitochondria. Moreover, Hela cells and 4T1 cells treated by CMMFTP showed strong red fluorescence under fluorescence microscopy, and clear cell images and cell edges, indicating that CMMFTP has good fluorescence imaging function and is expected to be a nano-fluorescent imaging agent.

#### 2.3.2. Intracellular GSH Depletion

TME is a complex environment in which numerous biomolecules affect ROS, especially GSH severely depletes ROS, so inhibition of ROS depletion by cellular GSH is an important strategy to improve the efficacy of cancer therapy. Previously, we examined the depletion of GSH by CMMFTP in solution and found that CMMFTP has a strong ability to deplete GSH. Based on the same principle, the intracellular GSH depletion ability of CMMFTP was examined using a GSH kit. The samples treated with different concentrations of CMMFTP were tested with a microplate reader, and the fluorescence intensity histogram was plotted as Figure 3D. When the concentrations of CMMFTP were 100 µg/mL, 200 µg/mL, and 400 µg/mL, about 40%, 55%, and 80% of intracellular GSH were consumed, respectively, indicating that CMMFTP has a strong ability to consume GSH in TME.

#### 2.3.3. Evaluation of Selective Cytotoxicity

CMMFTP cannot undergo Fenton-like reaction and produce ROS production under weak alkaline (pH 7.4) conditions without H_2_O_2_ and GSH, nor can it produce ^1^O_2_ in the dark. CMMFTP can produce ROS only under GSH activation in combination with acidic stimulation and light, thus CMMFTP is a smart and specific response ROS reactor under stimulation conditions that match the tumor microenvironment.

To test the specificity of CMMFTP on tumor cells, a comparative experiment was performed with L929, Hela, and 4T1 cells. A control group and six drug concentration gradients (12.5, 25, 50, 100, 200, 400 µg/mL), light and dark groups were set up and cell viability was analyzed by MTT method. Figure 3E showed the effect of CMMFTP on Hela cells. The cell survival rate decreased significantly with increasing drug concentration and incubation time, and reached 70% at 12.5 µg/mL for 4 h. Figure 3F shows that CMMFTP had no effect on L929 cells, and the cell viability was stable at 100% even after prolonged incubation at high concentrations (400 µg/mL, 24 h), indicating the excellent biocompatibility of CMMFTP. Therefore, it can be concluded that CMMFTP has a high ability to kill cancer cells with excellent biocompatibility. Figure 3G,H also shows the difference between Cu-MOFs and Cu-MOF@MnO_2_ in the presence and absence of light. The cell viability of the Cu-MOF + NIR group was 10% lower than that of the Cu-MOF (no light group) at a material concentration of 400 µg/mL. Apparently, the synergistic PDT and CDT effect increased the lethality of cancer cells. The cytotoxicity of the latter was higher than that of the former, indicating that the dual action of Cu(II) and MnO_2_ was stronger than that of Cu(II) alone. Moreover, the cell lethality in the Cu-MOF@MnO_2_ group was higher than that in the Cu-MOF group, apparently as a result of the dual CDT effect and the dual GSH depletion of Cu(II) and MnO_2_. The cell lethality rate in the CMMFTP group was significantly higher than that in the Cu-MOF@MnO_2_ group, suggesting that FA/TPP-induced tumor cell enrichment and precise mitochondrial anchoring further enhanced the antitumor effect. The same result was also observed on 4T1 cells (Figure 3I). Comparing Figure 3G,H, it was clear that the cell viability rate of the illuminated group was significantly lower than that of the unilluminated group, indicating that the synergistic effect of CDT/PDT was significantly better than CDT treatment alone, and the results on 4T1 cells were consistent with this.

#### 2.3.4. Evaluation of Intracellular ROS and Staining of Living and Dead Cells

**Figure 4:**
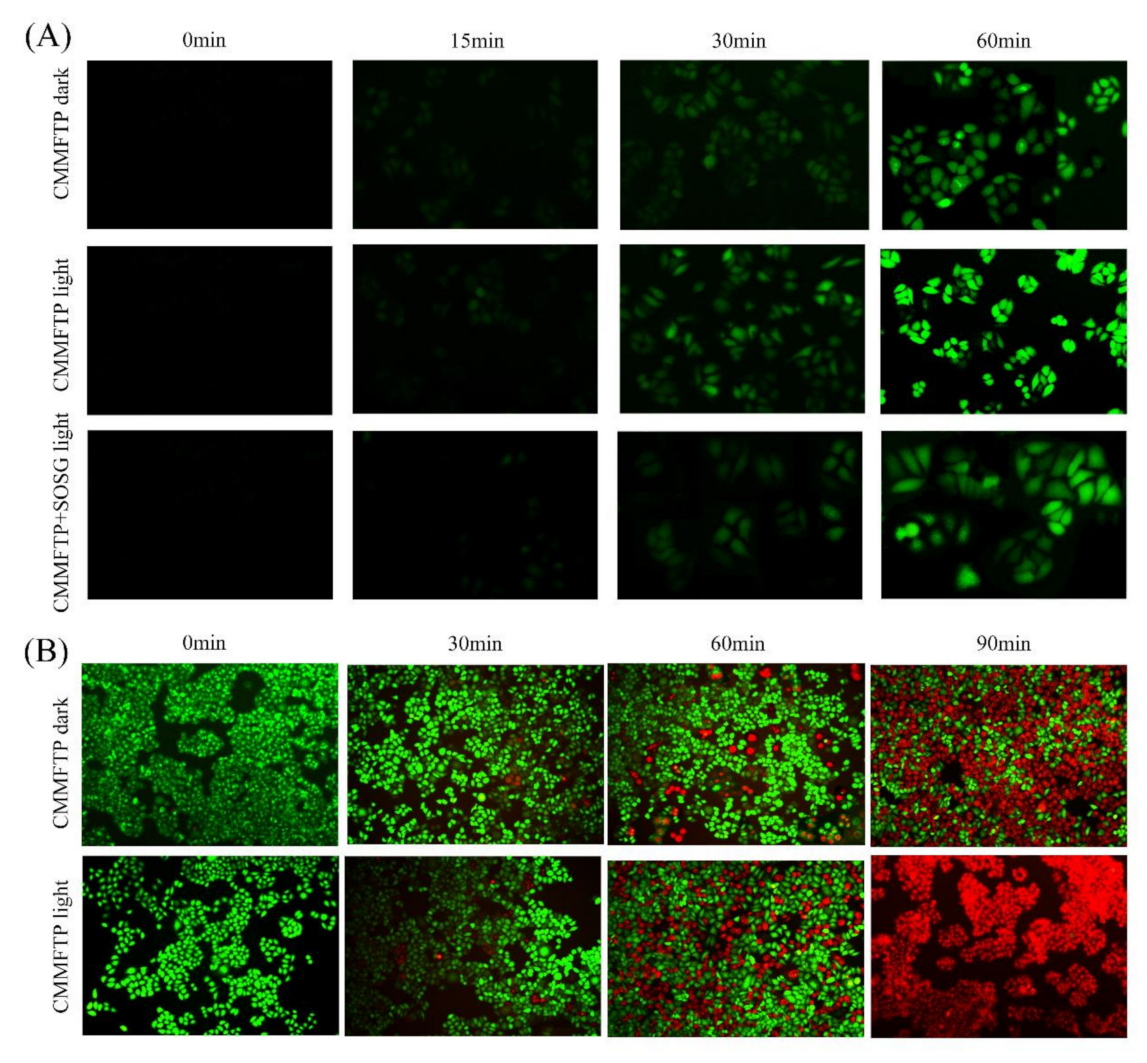
(**A**) Detection of intracellular ROS; (**B**) calcein-AM and PI co-staining of living and dead cells.

2,7-Dichlorodihydrofluorescein diacetate (DCFH-DA) is a universal oxidative stress indicator [46] for the evaluation of intracellular ROS levels (^•^OH, ^1^O_2_), and Singlet Oxygen Sensor Green (SOSG) is a specific probe for the evaluation of intracellular ^1^O_2_ levels. Hela cells were divided into three groups: CMMFTP + DCFH-DA dark, CMMFTP + DCFH-DA light, and CMMFTP + SOSG light. The cells were treated with drugs, incubated for 0–60 min and observed under a fluorescent inverted microscope. The results were shown in Figure 4A. ROS was produced quickly in the cells, with faint green fluorescence at 15 min, further enhanced at 30 min, and stronger at 60 min, indicating continuous intracellular ROS production within 0–60 min. The CMMFTP + SOSG + light group produced ^1^O_2_ in cells, indicating that CMMFTP has a PDT function in body. The CMMFTP + light group showed the strongest fluorescence, indicating that it produced much higher levels of ROS than the dark group, further highlighting the advantage of dual action of Fenton-like reaction and photosensitization reaction to produce ROS.

Calcein acetoxymethyl ester (calcein-AM) and propidium iodide (PI) double staining of cells was used to stain living and dead cells (Figure 4B). Calcein-AM can enter living cells and hydrolyze to produce calcein to fluoresce green (λ_ex_490/λ_em_515 nm), while PI can only enter the nucleus of dead cells and fluoresce red (λ_ex_490, 545/λ_em_617 nm). Hela cells were divided into two groups: CMMFTP dark and CMMFTP light, calcein-AM/PI double staining was added after administration, incubated for 0–90 min, and photographed under fluorescent inverted microscope. It was shown that dead cells began to appear after 30 min of administration and all cells in CMMFTP light group died at 90 min. The treatment effect of the light group was stronger than that of the dark group, again demonstrating the advantage of synergistic effect of CDT and PDT.

### 2.4. In Vivo Experiment

The anti-tumor activity tests of CMMFTP in vitro showed specific selectivity, modifiable smart selectivity, and outstanding synergistic CDT/PDT therapeutic advantages. Accordingly, Balb/c mice were used as animal models to investigate the in vivo antitumor activity of CMMFTP. Balb/c mice weighing 18–22 g at 6–7 weeks were randomly divided into three groups (5 mice in each group): saline group (blank control group), CMMFTP dark group, and CMMFTP light group.

**Figure 5:**
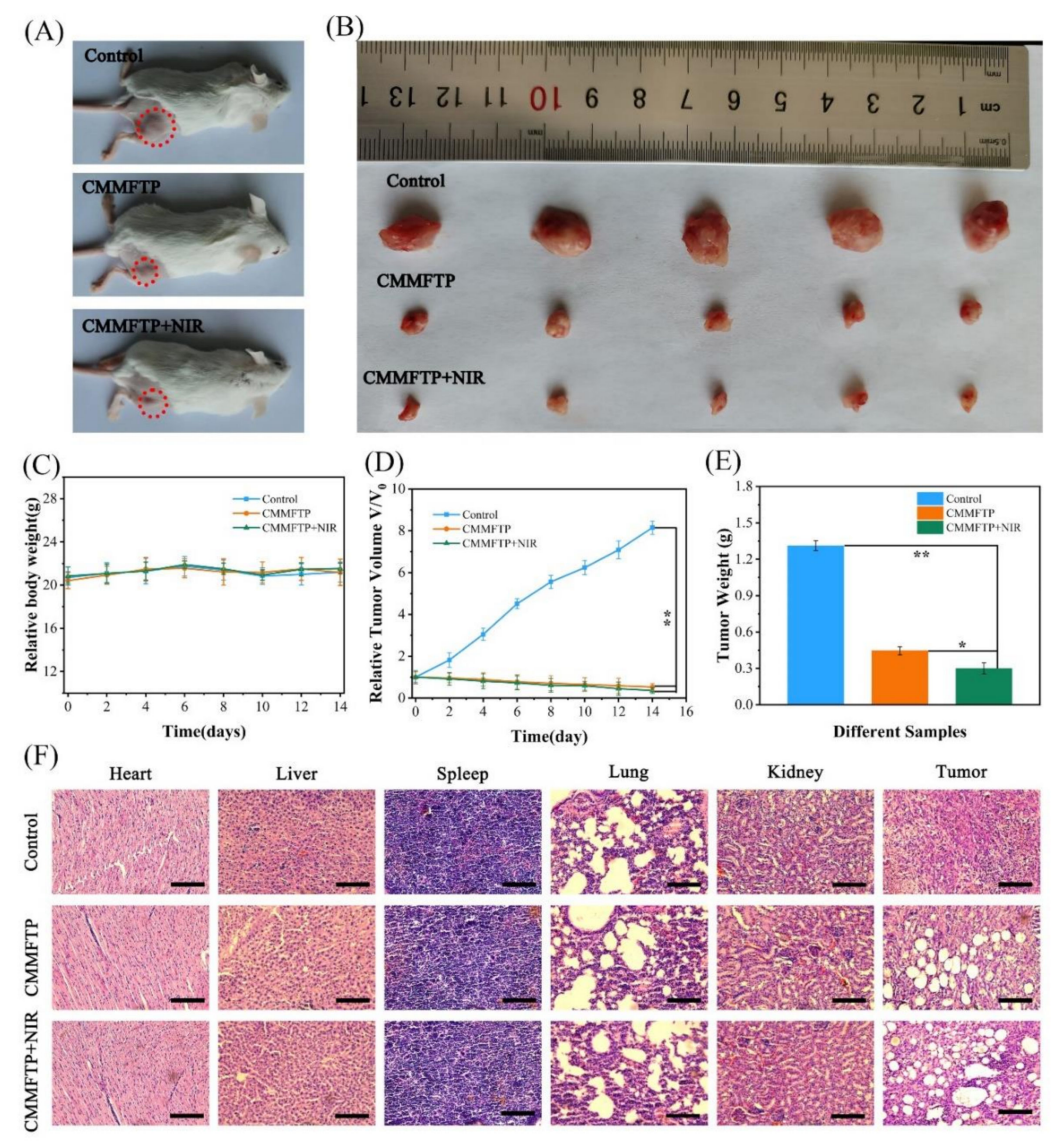
(**A**) Photographs of tumor-bearing mice in different experimental groups after 14 days of treatment; (**B**) images of tumors after different treatments; (**C**) changes in body weight of mice during treatment; (**D**) tumor volume and (**E**) tumor weight in different experimental groups after 14 days of treatment; (**F**) H&E staining images of heart, liver, spleen, lung, kidney, and tumors in different experimental groups after 14 days of treatment. Scale bar: 100 μm. ^∗^, *p* < 0.05; ^∗∗^, *p* < 0.01.

During the experiment, 4T1 cells were inoculated in the hindquarters of mice, and when the tumor volume reached about 100 mm^3^, the CMMFTP drug (200 μg/mL, 200 µL) was injected into the tail vein (except for the control group). After 6 h of drug administration, the CMMFTP light group was illuminated for 10 min with a light source wavelength of 660 nm and an optical density of 30 mW/cm^2^. The drug was administered again on day 7 (except for the control group) and the CMMFTP light group was again lighted. The mice were executed by cervical dislocation on day 14. The body weight and tumor volume of the mice were measured periodically during days 0–14. As shown in Figure 5A–E, the mice in the control group grew well with no major fluctuation in body weight and rapid growth of tumor volume, while the mice in the CMMFTP and CMMFTP light groups showed less fluctuation in body weight and gradual reduction in tumor volume, and the tumor sites were obviously crusted on the day after 14, indicating that CMMFTP had significant tumor growth inhibition and ablation effects. Visual estimation showed that the solid tumor volume in the CMMFTP dark group decreased by approximately 12% after two doses, while the solid tumor volume in the CMMFTP light group decreased by approximately 7%, indicating that CDT/PDT synergistic treatment significantly ablated solid tumors. The changes in tumor volume and mouse weight showed that CMMFTP had good tumor therapeutic effects and was biocompatible.

To investigate the effects of CMMFTP on tumor tissues and normal organs, heart, liver, spleen, lung, kidney, and tumor of mice were collected after 14 days of treatment and stained with hematoxylin and eosin (H&E) for histological study. As can be seen from Figure 5F, all organs in the CMMFTP dark and CMMFTP light groups were uniformly tightly organized with no obvious sites of damage, indicating that CMMFTP is a biocompatible nanomedicine with therapeutic selectivity and targeting. In contrast, the solid tumor tissues in the drug group were severely damaged, the tissue structure was relaxed, and there were obviously large white damage sites, especially in the CMMFTP light group, which showed larger damage area and the white damage spots, indicating that CMMFTP has a promising CDT/PDT synergistic anti-tumor effect and potential application in future.

## 3. Conclusions

In summary, this study successfully prepared a self-oxygenated ROS-enhanced metal-organic framework nanoreactor Cu-TCPP-MOFs@MnO_2_/FA/TPP@PAH (CMMFTP) based on specific CDT/PDT synergy and GSH depletion strategy to overcome the limitation of TME. The dual Fenton-like reaction and dual glutathione depletion by Cu-MOF and MnO_2_ greatly enhanced the antitumor effect in vitro and in vivo. CMMFTP achieved 96% growth inhibition of HeLa cells by synergistical PDT/CDT under NIR light excitation. The dual targeting of FA and TPP allows the nanoreactor to be precisely anchored in mitochondria, amplifying the antitumor effect. CMMFTP was biocompatible and presented 100% viability against normal cells even at high concentrations (400 µg/mL), while it was highly responsive to tumor cells and could be regulated by pH and GSH to generate ^•^OH and O_2_ by Fenton-like reactions. The design of this nanoreactor based on specific CDT/PDT synergy and precise targeting, as well as self-oxygenation to enhance ROS, provides new ideas for cancer therapy.

## Data Availability

Not applicable.

## Supplementary Materials

The following supporting information can be downloaded at: https://www.mdpi.com/article/10.3390/nano12142509/s1, Figure S1: DLS size of CMMFTP; Figure S2: Zeta potential of Cu-MOFs, Cu-MOF@MnO2, Cu-MOF@MnO2@FA/TPP, CMMFTP; Figure S3: Stability of CMMFTP in different solutions within 0–15 days; Figure S4: CMMFTP degrades MB under different concentration conditions (pH 5.5); Table S1: The absorbance and degradation rate of DPBF in CMMFTP and MB are at different.
