# Supplementary material for: Self-Supply Oxygen ROS Reactor via Fenton-like Reaction and Modulating Glutathione for Amplified Cancer Therapy Effect
Source: Nanomaterials (Basel). 2022 Jul 21;12(14):2509. doi: 10.3390/nano12142509 (PMC9319594; doi:10.3390/nano12142509)
Supplement: Supplementary file 1 [file nanomaterials-12-02509-s001.zip › nanomaterials-1790684-supplementary.pdf]

Supplementary Material

Self-Supply Oxygen ROS Reactor via Fenton-Like Reaction and Modulating Glutathione for Amplified Cancer Therapy Effect

Huanli Zhang †, Wei Ma †, Zhiqiang Wang, Xiaodan Wu, Hui Zhang, Wen Fang *, Rui Yan * and Yingxue Jin *

Key Laboratory for Photonic and Electronic Bandgap Materials, Ministry of Education, College of Chemistry & Chemical Engineering, Harbin Normal University, Harbin 150025, China; hxzhl0923@163.com (H.Z.); maweimcz@163.com (W.M.); wzq70402@163.com (Z.W.); smile_200325@163.com (X.W.); huihui279727690@163.com (H.Z.)

* Correspondence: fangwen06@163.com (W.F.); yanrui-1981@163.com (R.Y.); jyxprof@163.com (Y.J.)

† These authors contributed equally to the work.

1. Materials and Methods

1.1. Materials and Apparatus

Poly (allylamine hydrochloride) (PAH, MW 15000), $Cu(NO_3)_2$, N,N-Dimethylformamide (DMF), NaOH, $KMnO_4$, Folic acid (FA), triphenylphosphonium (TPP), meso-Tetra(4-carboxyphenyl)porphine (TCPP), $NaHCO_3$, H_2O_2 all purchased from Sinopharm Chemical Reagent Co., Ltd. (Shenyang, China). 1-Ethyl-3-(3-dimethylaminopropyl) carbodiimide hydrochloride (EDC), N-Hydroxysuccinimide (NHS), GSH, 5,5'-dithiobis(2-nitrobenzoic acid) (DTNB), polyvinyl pyrrolidone (PVP), $[Ru(dpp)_3]Cl_2$ (RDPP), 4,6-diamidino-2-phenylindole (DAPI), Mito-Tracker Green, Micro Reduced Glutathione (GSH) Assay Kit, Calcein acetoxymethyl ester (Calcein-AM), Propidium Iodide (PI), Singlet Oxygen Sensor Green (SOSG), 3-(4,5)-dimethylthiahiazo (-z-y1)-3,5-di-phenytetrazolium-romide (MTT) were purchased from Aladdin Industrial Co. (Shanghai, China). Fetal bovine serum (FBS), DMEM medium, 1640 medium, Phosphate Buffer (PBS), 1,3-diphenylisobenzofuran (DPBF), 5,5-dimethyl-1-Pyrroline-1-oxide (DMPO), 2,7-dichlorofluorescein diacetate (DCFH-DA), streptomycin, penicillium were purchased from Shanghai Yu Bo Biotecnology Co. Ltd (Shanghai China). Hela cell lines, 4T1 cell lines and L929 cell lines were provided by Harbin University of Commerce. Balb/c mice were provided by Harbin Medical University.

Transmission electron microscopy (TEM) and high-resolution transmission electron microscope (HR-TEM) images were obtained from a Tecnai G2 F200 S-TWIN. A 90Plus PALS zeta potential analyzer (Brookhaven, America) equipped with dynamic light scattering (DLS) was used to detect the NP size. The crystal structures of the obtained materials were characterized by X-ray diffraction (XRD, D5005, SIEMENS, Germany) with Kα radiation (λ=0.15405 nm) over the 2θ range of 5-80 °. UV-visible spectra were recorded with UV-2600 UV-vis spectrophotometer. Fluorescence emission and excitation spectra were performed on a Hitachi F-4600 spectrophotometer. Fourier Transform Infrared Spectrometer (FT-IR, Bruker Corporation, Germany) was used to evaluate the functional groups in all samples. X-ray photoelectron spectroscopy (XPS) was performed on ESCALAB 250Xi (Thermo Scientific). Photothermal and photodynamic effects were evaluated under the laser irradiation of 808 nm (Changchun New Industries Optoelectronics Technology Co. Ltd., China). EMX-plus Electron spin resonance spectroscopy (Germany Bruker). Bio Tek ELx800 Enzyme standard instrument (BioTek, USA).

1.2. Synthesis of CMMFTP

1.2.1. Cu-MOFs

The Cu-MOFs were prepared according to the literature (Angew Chem Int Ed Engl. 2016, 55, 6471-6475), briefly, $Cu(NO_3)_2$ (20 mg), PVP (100 mg), DMF (80 mL) and anhydrous ethanol (20 mL) were added to a 500 mL flask, stirred thoroughly, then TCPP (50 mg) was added and sonicated for 10 min. The solution was transferred to 250 mL stainless

steel autoclave and reacted at 80 °C for 3 h in the oven. After cooling to room temperature, the precipitate was collected by centrifugation (8000 rpm, 30 min), washed three times with ethanol and once with cold water, then freeze-dried for set aside.

1.2.2. Cu-MOFs@MnO_2

The Cu-MOFs@MnO_2 was prepared according to the method in the literature (J Am Chem Soc. 2019, 141, 11531-11539), in brief, 30 mg of Cu-MOFs and 30 mL of H_2O were added to a 100 mL beaker for ultrasonic treatment for 10 min, then PAH (40 mg in 15 mL of H_2O) was added and the pH was adjusted to neutral with $NaHCO_3$ saturated solution, and stirred at room temperature for 12 h. The precipitate was collected by centrifugation (8000 rpm, 15 min), washed 3 times with ethanol and once with cold water, then redispersed in 30 mL H_2O, sonicated for 10 min and dropped into $KMnO_4$ (20 mg in 5 mL of H_2O) under intense stirring. After dropping, the mixture was stirred for 30 min and then adjusted to weak acidity (pH 6-7) with 2 M HCl. The precipitation was collected by centrifugation (8000 rpm, 20 min), washed with cold water 3 times and freeze-dried to obtain Cu-MOFs@MnO_2.

1.2.3. Cu-MOFs@MnO_2/FA/TPP

30 mg Cu-MOF@MnO_2 and 30 mL DMF were added to a 100 mL beaker for ultrasonic dispersion for 10 min, then 144 mg EDC and 88 mg NHS were added for ultrasonic dispersion for 10 min, followed by stirring at room temperature for 12 h. The precipitate was collected by centrifugation (8000 rpm, 20 min), washed 3 times with ethanol and redispersed in 30 mL DMF. Then FA (30 mg) and TPP (20 mg) were added, sonicated for 30 min and stirred at 50 °C for 12 h. After the reaction, the precipitates were collected by centrifugation (8000 rpm, 20 min) and washed three times with ethanol and cold water respectively to obtain Cu-MOFs@MnO_2/FA/TPP.

1.2.4. CMMFTP

20 mg of Cu-MOFs@MnO_2/FA/TPP was dispersed in 20 mL of water and adjusted to weakly alkaline (pH 7.2) with $NaHCO_3$ saturated solution. Then 20 mg of PAH (in 10 mL of H_2O) was added, left overnight, the precipitate was collected by centrifugation (2000 rpm, 20 min), washed 3 times with cold water and freeze-dried to obtain CMMFTP.

1.3. Uv-Visible Absorption and Fluorescence Emission

The sample was prepared at the desired concentration, and the absorption spectrum and fluorescence spectrum were obtained by UV-visible spectrophotometer and fluorescence spectrophotometer, respectively.

1.4. Regulation of the Production •OH by pH and GSH

Prepared the solution with PBS: MB (10 μg/mL), GSH (5 μg/mL), CMMFTP (10, 20, 40, 60, 80 μg/mL), Cu-MOFs (40 μg/mL), Cu-MOFs@MnO_2 (40 μg/mL), H_2O_2 (30 μg/mL). Prepared samples: MB, MB+Cu-MOFs@MnO_2+H_2O_2 pH 7.4, MB+Cu-MOFs+H_2O_2 pH 6.8, MB+Cu-MOFs@MnO_2+H_2O_2 pH 6.8, MB+Cu-MOFs+H_2O_2+GSH pH 6.8, MB+Cu-MOFs@MnO_2+H_2O_2+GSH pH 5.5, MB+CMMFTP+H_2O_2+GSH pH 5.5. Sample preparation: MB+CMMFTP+H_2O_2+GSH pH 5.5 (CMMFTP 0, 10, 20, 40, 60, 80 μg/mL). The absorbance of the above samples was measured by UV-visible spectrophotometer.

DMPO was used as the trapping agent of hydroxyl radical. Cu-MOF and CMMFTP were tested. The concentration of H_2O_2 was 10 mM in a 10 μL dosage and the concentration of GSH was 0.67 mM in a 10 μL dosage. PBS was used as solvent, and DMPO was added to the samples and left for 30 min for ESR detection.

1.5. Regulation of the Production O_2 by pH and GSH

Monitoring O_2 production with RDPP, an O_2 sensing probe whose fluorescence is strongly quenched by O_2. Typically, 50 μL of RDPP ethanol solution (10×10^{-3} M) was added

to 1mL of sample solution (Cu-MOF, Cu-MOF@MnO2 and CMMFTP (20 μg/mL) solutions suspended in PBS (pH 5.5 or 7.4)) and the mixture was transferred to a colorimetric plate. After adding H_2O_2 (1.36 μg/mL), the fluorescence intensity of RDPP (λex=455nm) at 613 nm was recorded every 2 min.

1.6. Detection of $^1O_2$

Using DPBF as the reactive oxygen probe, the absorbance of DPBF after oxidation by ROS at 412 nm was measured by UV-visible spectrometer. CMMFTP and TCPP were used as the test object and the reference object was MB. The singlet oxygen yield of MB was 49%. PBS was used as solvent to prepare the sample to be tested so that its initial absorbance was 1. A 3 mL quartz cuvette was used to record the absorbance at 0-120 s.

SOSG captures $^1O_2$. As a specific probe of $^1O_2$, SOSG emits green fluorescence after oxidation by $^1O_2$, with an excitation wavelength of 490 nm and an emission wavelength of 525 nm. To test the material of CMMFTP, samples were made using 3 mL quartz cuvettes with PBS as solvent and 100 μL (5×10^{-6} M) of SOSG was added to each sample. After standing for 30 min, the fluorescence spectrum was measured.

1.7. Depletion GSH

Different concentrations of CMMFTP (0, 20, 50, 100 μg/mL) solutions were prepared in PBS and added into a colorimetric dish. H_2O_2 and GSH were added quantitatively as required in the experiment. After 30 min of standing, absorbance at 412 nm was recorded by UV-visible spectrophotometer.

1.8. In vitro Studies

1.8.1. Cell Incubation

The cells were cultured in medium containing 15% FBS and 1% penicillin-streptomycin solution in a 5% CO_2, 37 °C incubator. The cells of logarithmic growth stage were digested by 0.25% trypsin and those attached to the surface of the culture container were blown off to form a cell suspension and then inoculated. After inoculation, the cells were grown in a 5% CO_2 incubator at 37 °C for 24 h for later use.

1.8.2. Cell Uptake and Targeting

Hela cells and 4T1 cells were treated with CMMFTP (0-400 μg/mL) and incubated for 0-90 min, washed with PBS. Then the cells were fixed with 1 mL of 2.5% glutaraldehyde for 10 min, stained with DAPI (100 nM, 30 μL) for 10 min and washed with PBS. All cell samples were photographed under an inverted fluorescence microscope.

Hela cells and 4T cells were inoculated into 6-well plates at a density of 1.5×10^5 cells per well and divided into 2 groups: CMMFTP group, CMMFTP+FA group. Cells in each group were treated with the corresponding drug (200 μg/mL) and FA (20 μg/mL) and incubated in an incubator for 0-180 min. Then the cells were fixed with 1 mL 2.5% glutaraldehyde for 10 min, stained with DAPI (100 nM, 30 μL) for 10 min and Mito-Tracker Green (100 nM, 30 μL) for 30 min, washed 3 times with PBS. All cell samples were photographed under an inverted fluorescence microscope.

1.8.3. Intracellular GSH Depletion

Hela cells were inoculated into 96-well culture plates at a density of 6000 cells per well. The cells were then treated with different concentrations of CMMFTP (0-400 μg mL^{-1}) and co-incubated for 24 h. The cells were washed twice with PBS and collected by centrifugation. Subsequently, cells were suspended by adding 0.3-0.5 mL of PBS buffer and sonicated. Then the GSH content of these treated cells was measured using the Micro Reduced Glutathione (GSH) Assay Kit according to the manufacturer's instructions.

1.8.4. MTT assay

Cell viability was determined by MTT assay. Hela cells, L929 cells and 4T cells were seeded into 96-well plates, then the cells were incubated with 1 mL of DMEM containing CMMFTP, Cu-MOFs, Cu-MOFs@MnO_2 at different concentrations ranging from 12.5 to 400 μg/mL for 24 h and a blank control group treated with DMEM only was set up. After incubation, removed the original medium, PBS (200 μL) containing MTT (0.5 mg/mL) was added and incubation continued for 4 h. Then the PBS was replaced with DMSO (200 μL) and the mixture was shaken for 10 min to dissolve blue formazan. A microplate reader was used to measure the absorbance at 490 nm. Cell viability was calculated by comparing with the absorbance of the cells in control group.

1.8.5. Intracellular ROS Detection

The solution of CMMFTP (200 μg/mL) was prepared. Hela cells were seeded into culture dishes (2×10^5 cells per dish) for 24 h. Then the cells were incubated with 1 mL of DMEM containing CMMFTP. The cells were divided into three groups: CMMFTP+DCFH-DA dark group; CMMFTP+DCFH-DA light group; CMMFTP+SOSG light group. The cells were incubated in the incubator for 90 min. Then the medium was aspirated and discarded, washed 3 times with PBS, and observed with fluorescence inverted microscope. The concentration of DCFH-DA and SOSG were 0.5 μM.

1.8.6. Calcein AM/PI Double Staining of Living and Dead Cells

Cells were seeded into culture dishes (2×10^5 cells per dish) and incubated for 24 h. They were divided into two groups: the CMMFTP dark group and the CMMFTP light group, treated with CMMFTP (400 μg/mL, 1 mL) and incubated for 0-24 h. The 'CMMFTP light group' was irradiated with light (λ=660 nm, 30 W/cm^2) for 10 min after drug treatment and then incubated for 24 h. The cells were then stained with 100 μL of calcein-AM (2 μM) and PI (4.5 μM) for 30 min at 37 °C. The medium was discarded, washed 3 times with PBS, and then observed and photographed with fluorescence inverted microscope.

1.9. In vivo Experiment

Female Balb/c mice (6-7 weeks old, weighing 18-22 g) were used as animal models. Mice were injected subcutaneously with around 10^6 uterine cervical cancer cells (4T1). As the tumor volume of mice approached about 100 mm^3, the 10 mice were equally divided into three groups randomly and treated by tail vein injection with PBS (control group) or CMMFTP or CMMFTP+light (200 μg/mL, 200 μL) every 7 days for 2 weeks. For the CMMFTP+light group, after administration of the drug, irradiation with a laser (660 nm, 30 mW/cm^2). The body weight and tumor volume of each group were recorded every other day. The tumor volume was calculated according to the equation: tumor volume = (tumor length) × (tumor width)^2/2. On day 14, all mice were sacrificed by cervical dislocation, and the tumors were dissected and weighed. The fresh tumor tissues and normal tissues of the control and drug groups were fixed in 4% neutral buffered formalin, and then processed routinely into paraffin for hematoxylin and eosin (H&E) assay. They were examined under a digital microscope (Olympus).

2. The Absorbance and Degradation Rate of DPBF in CMMFTP and MB are at Different times

Table S1. The absorbance and degradation rate of DPBF in CMMFTP and MB are at different times.

Time(S)	DPBF（With CMMFTP）		DPBF（With MB）		DPBF（With TCPP）	
	Absorbance value（A）	$\ln(A_0/A_t)$	Absorbance value（A）	$\ln(A_0/A_t)$	Absorbance value（A）	$\ln(A_0/A_t)$
0	1.017	0	1.085	0	1.051	0
10	1.006	0.010875	1.052	0.030887	1.021	0.02896
20	0.981	0.03604	1.021	0.060797	0.983	0.066888
30	0.956	0.061854	0.994	0.087598	0.944	0.107371

40	0.933	0.086207	0.964	0.118244	0.897	0.158442
50	0.904	0.117783	0.915	0.170411	0.853	0.208738
60	0.877	0.148105	0.889	0.199238	0.811	0.259229
70	0.841	0.190021	0.847	0.247635	0.776	0.303345
80	0.812	0.225112	0.812	0.289835	0.742	0.348148
90	0.786	0.257656	0.761	0.354702	0.691	0.419358
100	0.759	0.292611	0.716	0.415655	0.657	0.469813
110	0.729	0.332939	0.682	0.464306	0.621	0.526166
120	0.703	0.369256	0.653	0.507758	0.583	0.58931

3. DLS Size of CMMFTP

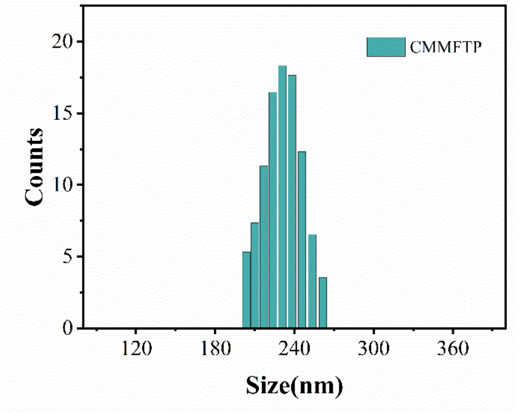

Figure S1. DLS size of CMMFTP.

4. Zeta Potential of Cu-MOFs, Cu-MOF@MnO$_2$,Cu-MOF@MnO$_2$@FA/TPP, CMMFTP

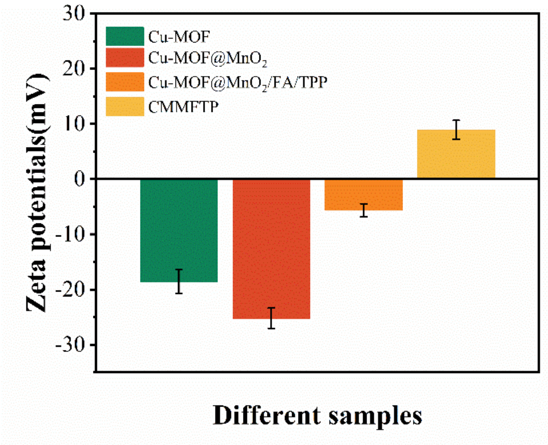

Figure S2. Zeta potential of Cu-MOFs, Cu-MOF@MnO$_2$, Cu-MOF@MnO$_2$@FA/TPP, CMMFTP.

5. Stability of CMMFTP in Different Solutions within 0-15 days

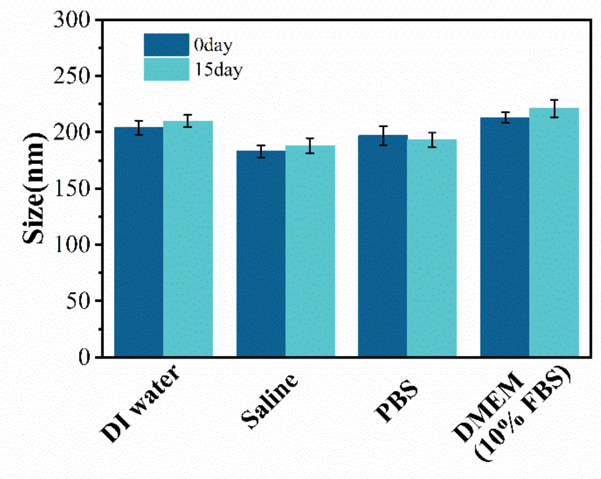

Figure S3. Stability of CMMFTP in different solutions within 0-15 days.

6. CMMFTP Degrades MB under Different Concentration Conditions (pH 5.5)

Sample 1: CMMFTP(0 μg/mL)+MB+H_2O_2+GSH pH 5.5
Sample 2: CMMFTP(10 μg/mL)+MB+H_2O_2+GSH pH 5.5
Sample 3: CMMFTP(20 μg/mL)+MB+H_2O_2+GSH pH 5.5
Sample 4: CMMFTP(40 μg/mL)+MB+H_2O_2+GSH pH 5.5
Sample 5: CMMFTP(60 μg/mL)+MB+H_2O_2+GSH pH 5.5
Sample 6: CMMFTP(80 μg/mL)+MB+H_2O_2+GSH pH 5.5

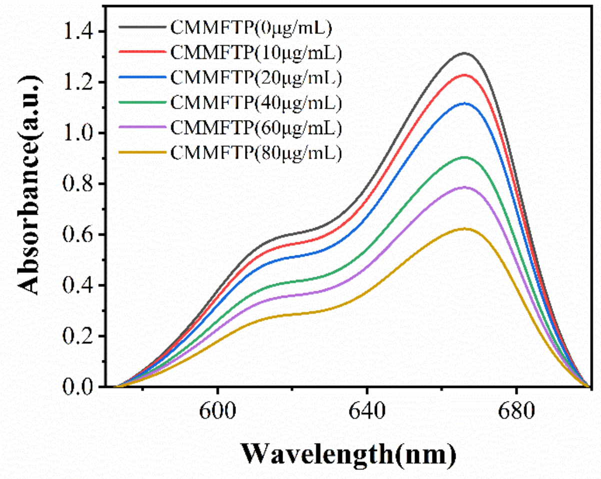

Figure S4. CMMFTP degrades MB under different concentration conditions (pH5.5).
